# Supplementary material for: Massively parallel reporter assay for mapping gene-specific regulatory regions at single-nucleotide resolution
Source: eLife. 2026 Feb 25;14:RP107565. doi: 10.7554/eLife.107565 (PMC12935429; doi:10.7554/eLife.107565)
Supplement: Supplementary file 1. — (a) PCR primers used in all experiments. (b) Genomic coordinates for all regions of interest. (c–g) Quantification of EGFP expression from electroporated plasmid constructs and colocalization with cell-type-specific gene expression. [file elife-107565-supp1.docx]

| **Supplementary File 1a. Primer sequences** | | |
| --- | --- | --- |
| **Method** | **Primer Name** | **Sequence** |
| Stagintbc7 vector design | bc7_ins1 | GGCGCGCCaGATCGGAAGAGCACACGTCTGAACTCCAGTCACCCTTACTTGTACAGCTCGTCCATG |
|  | bc7_ins2 | CCGATCtGGCGCGCCSWSWSWSWSWSWSWSWSWSWSWSWaGATCGGAAGAGCGTCGTGTAGGGAAAGAGTGTCCGGCCCCTTGAGCATCT |
|  | bc7_ins1A | GCCTGTATGGCGCGCCaGATCGGAAGAGCACACGTCTGAACTCCAGTCACCCTTACTTGTACAGCTCGTCCATG |
|  | bc7_ins1B | CTTGCACAGGCGCGCCaGATCGGAAGAGCACACGTCTGAACTCCAGTCACCCTTACTTGTACAGCTCGTCCATG |
|  | bc7_ins1C | TCTTCGTGGGCGCGCCaGATCGGAAGAGCACACGTCTGAACTCCAGTCACCCTTACTTGTACAGCTCGTCCATG |
|  | bc7_ins1D | AGGTTCACGGCGCGCCaGATCGGAAGAGCACACGTCTGAACTCCAGTCACCCTTACTTGTACAGCTCGTCCATG |
|  | bc7_ins2A | CCGATCtGGCGCGCCATACAGGCVHBDVHBDVHBDVHBDVHBDVHBDaGATCGGAAGAGCGTCGTGTAGGGAAAGAGTGTCCGGCCCCTTGAGCATCT |
|  | bc7_ins2B | CCGATCtGGCGCGCCTGTGCAAGVHBDVHBDVHBDVHBDVHBDVHBDaGATCGGAAGAGCGTCGTGTAGGGAAAGAGTGTCCGGCCCCTTGAGCATCT |
|  | bc7_ins2C | CCGATCtGGCGCGCCCACGAAGAVHBDVHBDVHBDVHBDVHBDVHBDaGATCGGAAGAGCGTCGTGTAGGGAAAGAGTGTCCGGCCCCTTGAGCATCT |
|  | bc7_ins2D | CCGATCtGGCGCGCCGTGAACCTVHBDVHBDVHBDVHBDVHBDVHBDaGATCGGAAGAGCGTCGTGTAGGGAAAGAGTGTCCGGCCCCTTGAGCATCT |
| LS-MPRA Adapters | enh7_5'1S | CGATAGTCGACCAATTGCTCGAGGGTGACTGGAGTTCAGACGTGTGCTCTTCCGATC*t |
|  | enh7_5'2phos | /5Phos/GATCGGAAGAGCACACGTCTGAACTCCAGTCACCCTCGAGCAATTGGTCGACTATCG |
|  | enh7_3'1phos | /5Phos/GGCGCGCCattaaGCTGGTAGACTgcgatCT |
|  | enh7_3'2S | AGatcgcAGTCTACCAGCttaatGGCGCGCC*t |
| Amplify fragments | enh7_ampF | CGATAGTCGACCAATTGCTC |
|  | enh7_ampR | AGatcgcAGTCTACCAGC |
| d-MPRA assembly | Olig2_NR1_mutF | CGATAGTCGACCAATTGCTCGAcagacatcaaatcagtaagccctgttg |
|  | Olig2_NR1_mutR | atcgcAGTCTACCAGCttaatTTGTTAGTGGCGTCTGTCCCG |
|  | Olig2_NR2_mutF | CGATAGTCGACCAATTGCTCGAgagtggtcctcacatgcccaa |
|  | Olig2_NR2_mutR | atcgcAGTCTACCAGCttaatCGTGTTTCCCAGCTGGCT |
|  | Olig2_NR3_mutF | CGATAGTCGACCAATTGCTCGActaggtgcttggaccccatct |
|  | Olig2_NR3_mutR | atcgcAGTCTACCAGCttaatAGTTGAGGCTTGGGATTCCGG |
|  | MutF | CGATAGTCGACCAATTGCTCGA |
|  | MutR | atcgcAGTCTACCAGCttaat |
|  | StatadualA_gfpF | GCTGGTAGACTgcgatAcctcaCTATATAATGGAAGCTTG |
|  | StatadualA_gfpR | CCTCGAGCAATTGGTCGACTATCGATTACTTGTACAGCTCGTCCATGCCG |
| LS-MPRA BC sequencing | bc7_cdnaR | AAGTCAGATGCTCAAGGGGC |
|  | bc7_cdnaF | GCAGAAGAACGGCATCAAGG |
|  | bc7_cdnaR2 | GCCGGACACTCTTTCCCTAC |
| d-MPRA BC sequencing | bc7_cdnaR_WPRE | AGCAGCGTATCCACATAGCG |
|  | bc7_cdnaF* | GCAGAAGAACGGCATCAAGG |
|  | MutR* | atcgcAGTCTACCAGCttaat |
|  | dualmut_IllR1F | ACACTCTTTCCCTACACGACGCTCTTCCGATCtACCAATTGCTCGAGG |
|  | dualmut_IllR2F | ACACTCTTTCCCTACACGACGCTCTTCCGATCTACCAATTGCTCGACA |
|  | dualmut_IllR2R | GTGACTGGAGTTCAGACGTGTGCTCTTCCGATCtgcAGTCTACCAGCttaat |
| Construct vector | compvec_F | CAGCTCACTCAAAGGCGGTA |
|  | compvec_R | CCTCGAGCAATTGGTCGACTATC |
| Olig2-NR1 and -NR2 constructs | Olig2_NR1F | GACCAATTGCTCGAGGcagacatcaaatcagtaagccctgttg |
|  | Olig2_NR1R | ATGTGCTTTTttgttagtggcgtctgtcccg |
|  | Olig2_NR2F | GACCAATTGCTCGAGGgagtggtcctcacatgccc |
|  | Olig2_NR2R | GGAACAATGTGCTTTTcgtgtttcccagctggc |
|  | Olig2_minpr-GFPint | AAAAGCACATTGTTCCCGCC |
|  | GFPint_vecR | GCCTTTGAGTGAGCTGgccctcccatatgtccttcc |
| Olig2-NR3 construct | Olig2_NR3F | GACATATGGGAGGGCCTAGGTGCTTGGACCCCATC |
|  | Olig2_NR3R | GCCTTTGAGTGAGCTGagttgaggcttgggattccg |
|  | Vec_GFPintF | gaccaattgctcgaggAAAAGCACATTGTTCCCGCC |
|  | GFPint_R | GCCCTCCCATATGTCCTTCC |
| Olig2-composite construct | Olig2_NR1F* | GACCAATTGCTCGAGGcagacatcaaatcagtaagccctgttg  |
|  | Olig2comp_NR1R | GGACCACTCttgttagtggcgtctgtccc |
|  | Olig2comp_NR2F | cactaacaaGAGTGGTCCTCACATGCCCa |
|  | Olig2_NR2R* | GGAACAATGTGCTTTTcgtgtttcccagctggc |
|  | Olig2_minpr-GFPint* | AAAAGCACATTGTTCCCGCC |
|  | GFPint_R* | GCCCTCCCATATGTCCTTCC |
|  | Olig2_NR3F* | GACATATGGGAGGGCCTAGGTGCTTGGACCCCATC |
|  | Olig2_NR3R* | GCCTTTGAGTGAGCTGagttgaggcttgggattccg |
| Backbone control constructs | Vec_GFPintF* | gaccaattgctcgaggAAAAGCACATTGTTCCCGCC |
|  | Vec_nominprGFPF | accaattgctcgaggGCCACCATGGTGAGCAAG |
|  | GFPint_R* | GCCCTCCCATATGTCCTTCC |
| Ngn2-CRM1, -CRM2, and  -CRM3 constructs | Ngn2-CRM1_F | ACCAATTGCTCGAGGgaagattgggagtatgaatagttgtc |
|  | Ngn2-CRM1_R | atcgcAGTCTACCAGCaatgctcacatcctccctcag |
|  | Ngn2-CRM2_F | ACCAATTGCTCGAGGtctcatcagtcccaaaactggc |
|  | Ngn2-CRM2_R | atcgcAGTCTACCAGCaatgatggtgggatggagag |
|  | Ngn2-CRM3_F | ACCAATTGCTCGAGGactcgggcccctaatgagc |
|  | Ngn2-CRM3_R | atcgcAGTCTACCAGCtttatctaatcagcataaaatggttctaaagctcc |
|  | TATAminpr_F | GCTGGTAGACTgcgatCT |
|  | GFPint_vecR | GCCTTTGAGTGAGCTGgccctcccatatgtccttcc |
| Ngn2-CRM4 construct | Ngn2-CRM4_F | GACATATGGGAGGGCcctaaaagtgagaagcctggag |
|  | Ngn2-CRM4_R | GCCTTTGAGTGAGCTGcaaggaggcaataaaccccc |
|  | Vec_TATAminpr_F | GACCAATTGCTCGAGGGCTGGTAGACTgcgatCT |
|  | GFPint_R* | GCCCTCCCATATGTCCTTCC |
| cOLIG2-CRM1 construct | cOLIG2_CRM1F | ACCAATTGCTCGAGGaccaaagagttaatttgcgtttttaaacc |
|  | cOLIG2_CRM1R | CTTTATAGCCGCGCCGGGctgctgtaatgtttttgttatttttcctc |
|  | cOLIG2_promF | cccggcgcggctataaaggc |
|  | cOLIG2_promR | CTCACCATGGTGGCggctctcaccgtgctcgggg |
|  | GFPint_F | GCCACCATGGTGAGCAAG |
|  | GFPint_vecR* | GCCTTTGAGTGAGCTGGCCCTCCCATATGTCCTTCC |
| cOLIG2-CRM2 construct | cOLIG2_CRM2F | ACCAATTGCTCGAGGgttacgcgttacgatggatctgac |
|  | cOLIG2_promR* | CTCACCATGGTGGCggctctcaccgtgctcgggg |
|  | GFPint_F* | GCCACCATGGTGAGCAAG |
|  | GFPint_vecR* | GCCTTTGAGTGAGCTGGCCCTCCCATATGTCCTTCC |
| cOLIG2-CRM3 construct | cOLIG2_CRM3F | GACATATGGGAGGGCgctcccattggtcagcgctg |
|  | cOLIG2_CRM3R | GCCTTTGAGTGAGCTGagcccgtaatcccgcgc |
|  | cOLIG2-vec_promF | ACCAATTGCTCGAGGcccggcgcggctataaaggc |
|  | cOLIG2_promR* | CTCACCATGGTGGCggctctcaccgtgctcgggg |
|  | GFPint_R* | GCCCTCCCATATGTCCTTCC |
| ddPCR | Olig2_qF | GCGAGCACCTCAAATCTAATTC |
|  | Olig2_qR | AAAAGATCATCGGGTTCTGGG |
|  | Hprt_qF | CCCCAAAATGGTTAAGGTTGC |
|  | Hprt_qR | AACAAAGTCTGGCCTGTATCC |
|  | *reused |  |

| **Supplementary File 1b: Genome Coordinates for ROIs** | | | | |
| --- | --- | --- | --- | --- |
| Genome | Chromosome | Start | End | Description |
| mm10 | chr6 | 115930332 | 115930431 | Rho PPR (Zack et al., 1991) |
|  | chr6 | 115931731 | 115931977 | Rho RER (Nie et al., 1996) |
|  | chr6 | 115928742 | 115929214 | Rho CBR (Corbo et al., 2010) |
|  | chr11 | 50842558 | 50842758 | Grm6 CRM (Kim et al., 2008) |
|  | chr12 | 84567682 | 84569914 | Vsx2 2.4 kb promoter CRM (Rowan et al., 2005) |
|  | chr12 | 84552103 | 84552267 | Vsx2 164 bp enhancer CRM (Kim et al., 2008) |
|  | chr12 | 84532102 | 84532711 | Vsx2 ECR4 homologue (Buenaventura et al., 2018) |
|  | chr12 | 84532049 | 84533013 | Vsx2 CRC-SE-mR0-37 (Honnell et al., 2022) |
|  | chr12 | 84550173 | 84553991 | Vsx2 CRC-SE-mR3-17 (Honnell et al., 2022) |
|  | chr7 | 13397865 | 13398310 | Cabp5 CRM (Kim et al., 2008) |
|  | chr16 | 91140523 | 91140769 | Olig2-NR1 |
|  | chr16 | 91223668 | 91223849 | Olig2-NR2 |
|  | chr16 | 91232270 | 91232482 | Olig2-NR3 |
|  | chr16 | 91225474 | 91225600 | Olig2 minimal promoter |
|  | chr16 | 91235256 | 91238546 | Olig2-K23 (Sun et al., 2006) |
|  | chr16 | 91139504 | 91141503 | Olig2-ULTRA (Chen et al., 2008) |
|  | chr16 | 91223550 | 91225549 | Olig2-basal promoter (Chen et al., 2008) |
|  | chr16 | 91191767 | 91193766 | Olig2-EC2 (Fan et al., 2023) |
|  | chr16 | 91208209 | 91211318 | Olig2-5F7 (Friedli et al., 2010) |
|  | chr3 | 127628497 | 127632832 | Ngn2-TgN2 (Simmons et al., 2001) |
|  | chr3 | 127642407 | 127642608 | Ngn2-CRM1 |
|  | chr3 | 127605638 | 127605854 | Ngn2-CRM2 |
|  | chr3 | 127632596 | 127632883 | Ngn2-CRM3 |
|  | chr3 | 127572578 | 127572810 | Ngn2-CRM4 |
|  |  |  |  |  |
| galGal6 | chr1 | 106522687 | 106522790 | cOLIG2 minimal promoter |
|  | chr1 | 106486012 | 106486309 | cOLIG2-CRM1 |
|  | chr1 | 106522473 | 106522687 | cOLIG2-CRM2 |
|  | chr1 | 106531687 | 106531776 | cOLIG2-CRM3 |

| **Supplementary File 1c. Quantification of GFP and Olig2 co-localization in embryonic mouse retina** | | | | | | |
| --- | --- | --- | --- | --- | --- | --- |
| **Plasmid** | **Electroporated Olig2+ cells that express GFP protein (%)** | | **GFP protein+ cells that express Olig2 (%)** | | **Electroporated Olig2-cells that express GFP protein (%)** | |
|  | **GFP+** | **GFP-** | **Olig2+** | **Olig2-** | **GFP+** | **GFP-** |
| Olig2-NR1 | 100 | 0 | 15.6 | 84.4 | 66.8 | 33.2 |
| Olig2-NR2 | 100 | 0 | 29.7 | 70.3 | 48.6 | 51.4 |
| Olig2-NR3 | 67.9 | 32.1 | 17.0 | 83.0 | 50.2 | 49.8 |
| Olig2-Triple Composite | 100 | 0 | 21.4 | 78.6 | 62.1 | 37.9 |

| **Supplementary File 1d. Quantification of CRM-directed RNA and *Olig2* RNA or protein** | | | | | | | | | | |
| --- | --- | --- | --- | --- | --- | --- | --- | --- | --- | --- |
| **Plasmid** | **Electroporated**  ***Olig2* RNA+ cells that expressed GFP (%)** | | | | ***GFP* RNA+ cells that expressed *Olig2* RNA (%)** | | **GFP protein+ cells that expressed *Olig2* RNA (%)** | | ***Olig2* RNA-cells that expressed**  **GFP (%)** | |
|  | ***GFP* RNA only** | ***GFP* RNA & protein** | **GFP protein only** | **GFP-** | ***Olig2* RNA+** | ***Olig2* RNA-** | ***Olig2* RNA+** | ***Olig2* RNA-** | **GFP+** | **GFP-** |
| Olig2-NR1 | 19.3 | 49.8 | 30.2 | 0.64 | 52.6 | 47.4 | 40.6 | 59.4 | 57.7 | 42.3 |
| Olig2-NR2 | 35.6 | 45.5 | 10.9 | 8.0 | 64.5 | 35.5 | 38.8 | 61.2 | 27.9 | 72.1 |
| Olig2-NR3 | 34.0 | 5.1 | 0.0 | 60.9 | 39.6 | 60.4 | 35.7 | 64.3 | 5.5 | 94.5 |
| Olig2-Triple Composite | 14.2 | 55.0 | 25.0 | 5.8 | 63.3 | 36.7 | 46.0 | 54.0 | 63.5 | 36.5 |

| **Supplementary File 1e. Quantification of CRM-directed RNA and *Ngn2* RNA or protein** | | | | | | | | | | |
| --- | --- | --- | --- | --- | --- | --- | --- | --- | --- | --- |
| **Plasmid** | **Electroporated**  ***Ngn2* RNA+ cells that expressed GFP (%)** | | | | ***GFP* RNA+ cells that expressed *Ngn2* RNA (%)** | | **GFP protein+ cells that expressed *Ngn2* RNA (%)** | | ***Ngn2* RNA-cells that expressed**  **GFP (%)** | |
|  | ***GFP* RNA only** | ***GFP* RNA & protein** | **GFP protein only** | **GFP-** | ***Ngn2* RNA+** | ***Ngn2* RNA-** | ***Ngn2* RNA+** | ***Ngn2* RNA-** | **GFP+** | **GFP-** |
| Ngn2-CRM1 | 22.2 | 30.6 | 3.7 | 43.5 | 6.6 | 93.4 | 6.5 | 93.5 | 48.2 | 51.8 |
| Ngn2-CRM2 | 7.3 | 86.4 | 3.3 | 2.9 | 20.7 | 79.3 | 19.7 | 80.3 | 88.8 | 11.2 |
| Ngn2-CRM3 | 32.9 | 59.6 | 4.4 | 3.1 | 38.5 | 61.5 | 32.3 | 67.7 | 69.3 | 30.7 |
| Ngn2-CRM4 | 9.4 | 0 | 0 | 90.6 | 11.9 | 88.1 | 10.6 | 89.4 | 14.8 | 85.2 |
| Ngn2-CRM3 (reduced ex vivo culture) | 24.8 | 60.2 | 7.7 | 7.2 | 49.2 | 50.8 | 44.0 | 56.0 | 39.3 | 60.7 |

| **Supplementary File 1f. Quantification of CRM-directed RNA and chick *OLIG2* RNA or protein** | | | | | | | | | | |
| --- | --- | --- | --- | --- | --- | --- | --- | --- | --- | --- |
| **Plasmid** | **Electroporated**  ***OLIG2* RNA+ cells that expressed GFP (%)** | | | | ***GFP* RNA+ cells that expressed *OLIG2* RNA (%)** | | **GFP protein+ cells that expressed *OLIG2* RNA (%)** | | ***OLIG2* RNA-cells that expressed**  **GFP (%)** | |
|  | ***GFP* RNA only** | ***GFP* RNA & protein** | **GFP protein only** | **GFP-** | ***OLIG2* RNA+** | ***OLIG2* RNA-** | ***OLIG2* RNA+** | ***OLIG2* RNA-** | **GFP+** | **GFP-** |
| cOLIG2-CRM1 | 5.5 | 90.3 | 1.7 | 2.5 | 45.7 | 54.3 | 45.3 | 54.7 | 68.2 | 31.8 |
| cOLIG2-CRM2 | 4.1 | 90.9 | 3.5 | 1.5 | 34.5 | 65.5 | 32.5 | 67.5 | 74.6 | 25.4 |
| cOLIG2-CRM3 | 1.0 | 0 | 0 | 98.9 |  |  |  |  | 0.4 | 99.6 |

| **Supplementary File 1g. Quantification of GFP and Olig2 co-localization in postnatal mouse retina** | | | | | | |
| --- | --- | --- | --- | --- | --- | --- |
| **Plasmid** | **Electroporated Olig2+ cells that express GFP protein (%)** | | **GFP protein+ cells that express Olig2 (%)** | | **Electroporated Olig2-cells that express GFP protein (%)** | |
|  | **GFP+** | **GFP-** | **Olig2+** | **Olig2-** | **GFP+** | **GFP-** |
| Olig2-NR1 | 99.5 | 0.5 | 88.0 | 12.0 | 68.0 | 32.0 |
| Olig2-NR2 | 97.9 | 2.1 | 85.3 | 14.7 | 51.1 | 48.9 |
| Olig2-NR3 | 95.8 | 4.2 | 70.8 | 29.2 | 56.2 | 43.8 |
| Olig2-Triple Composite | 99.8 | 0.2 | 80.9 | 19.1 | 65.8 | 34.2 |
