# Supplementary material for: Massively parallel reporter assay for mapping gene-specific regulatory regions at single-nucleotide resolution
Source: eLife. 2026 Feb 25;14:RP107565. doi: 10.7554/eLife.107565 (PMC12935429; doi:10.7554/eLife.107565)
Supplement: Source data 1. [file elife-107565-data1.pdf]

Association Library characteristics							
**Gene-of-interest**	**Cabp5**	**Grm6**	**Rho**	**Vsx2**	**Olig2**	**Neurog2**	**cOLIG2**
**BAC clone**	RP23-125H22	RP23-417M10	RP23-219M6	RP23-127O21	CH29-613L21	RP23-182M12	CH261-60J3
**Size of clone (bp)**	175,529	196,198	205,479	224,092	198,802	176,837	234,491
**Sequenced reads**	32,993,015	35,980,528	33,804,998	38,542,527	99,453,803	123,039,602	104,836,048
**Unique fragments sequenced**	6,742,335	6,342,682	6,528,168	5,065,113	7,273,814	9,236,041	11,579,155
**Predicted total unique fragments (Vmax)**	9,349,334	8,328,615	8,750,444	6,151,434	7,882,427	10,090,748	13,254,886
**Estimated under-sequencing (fragments)**	27.9%	23.8%	25.4%	17.7%	7.7%	8.5%	12.6%
**Unique barcode–fragment pairings**	9,283,048	8,115,758	8,339,844	7,121,265	17,931,082	27,383,903	44,774,642
**Unique barcodes sequenced**	2,745,009	3,299,572	3,434,846	3,541,130	6,494,202	7,894,680	6,859,915
**Predicted total unique barcodes (Vmax)**	3,033,326	3,694,093	3,902,617	3,991,963	6,911,736	8,419,118	7,213,082
**Estimated under-sequencing (barcodes)**	9.5%	10.7%	12.0%	11.3%	6.0%	6.2%	4.9%
**Mean barcodes per fragment±SD**	1.38±0.91	1.28±0.73	1.28±0.73	1.41±0.85	2.47±3.23	2.96±3.51	3.87±4.06
**Fragments with 1 BC (%)**	5,114,287 (75.9)	5,091,388 (80.3)	5,270,065 (80.7)	3,719,724 (73.4)	3,417,706 (47.0)	3,959,601 (42.9)	3,734,149 (32.2)
**Barcode Collisions (%)**	1,735,600 (63.2)	1,794,631 (54.4)	1,848,433 (53.8)	1,674,020 (47.3)	3,916,149 (60.3)	5,250,728 (66.5)	5,080,559 (70.1)

Fragment size distribution plots

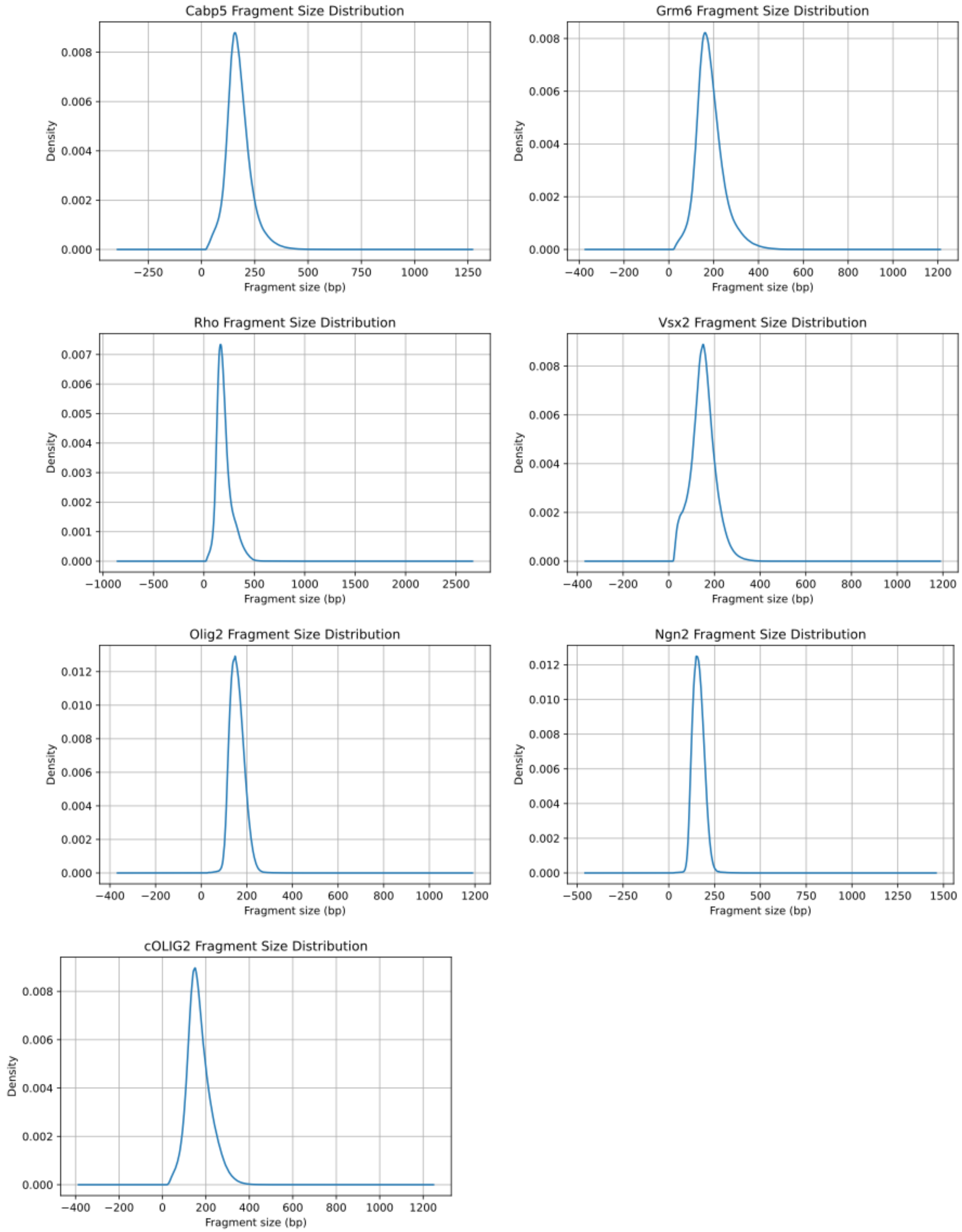

Correlation between fragment size and activity plots

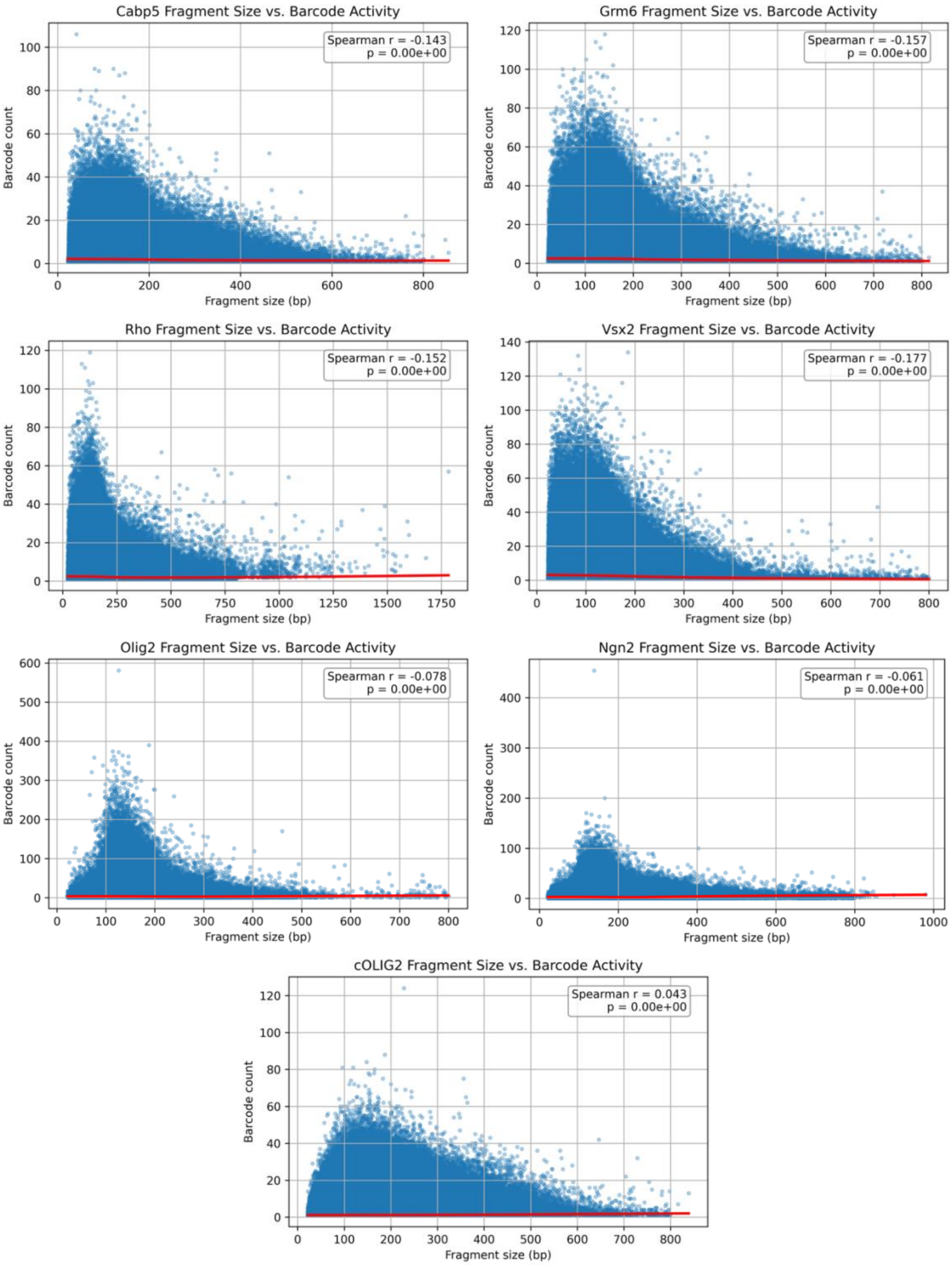

Barcode and sequence saturation curves

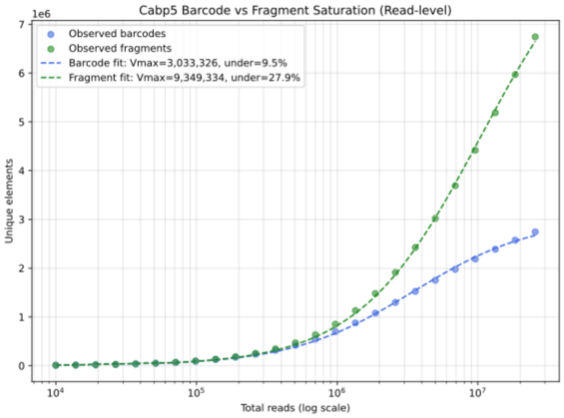

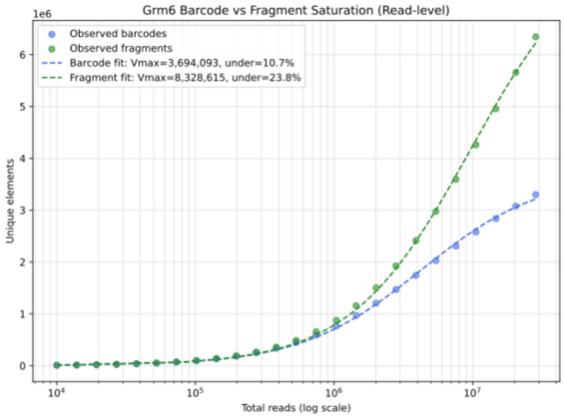

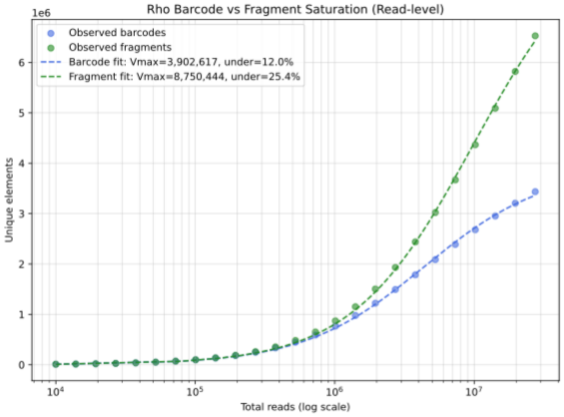

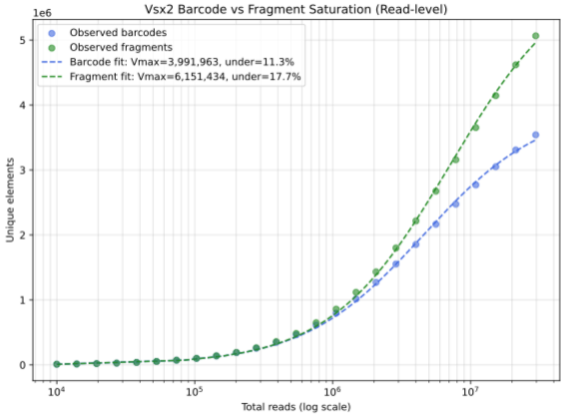

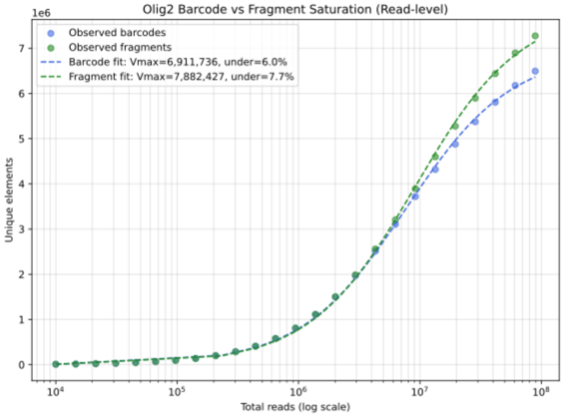

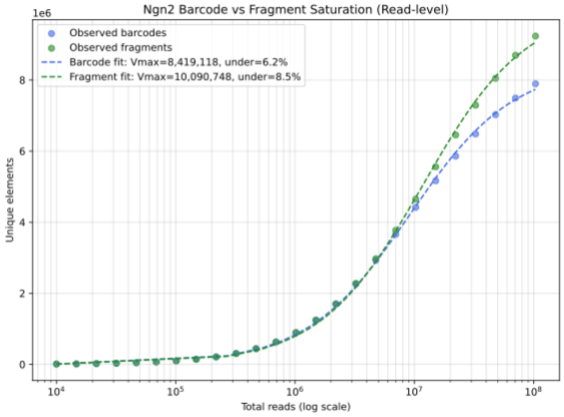

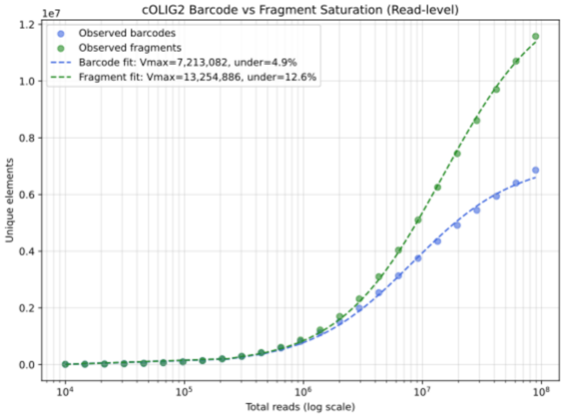

Unique barcode-fragment pair saturation curves

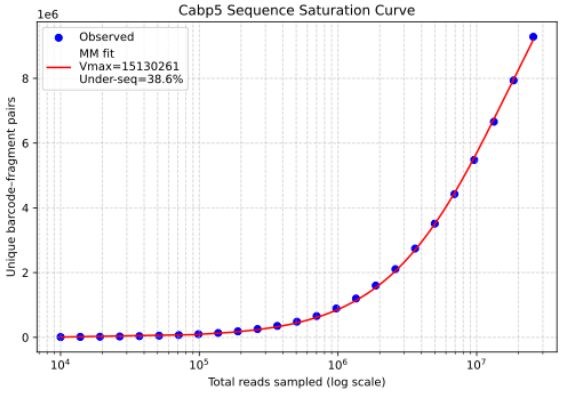

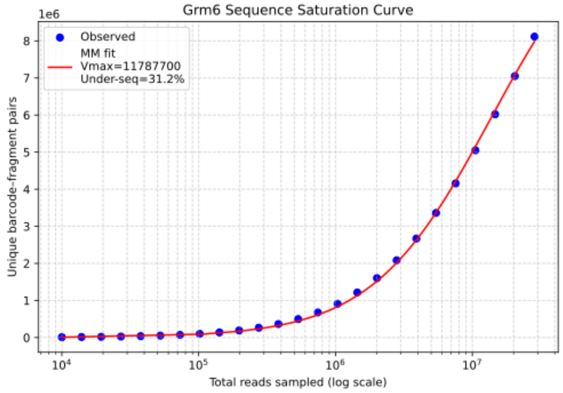

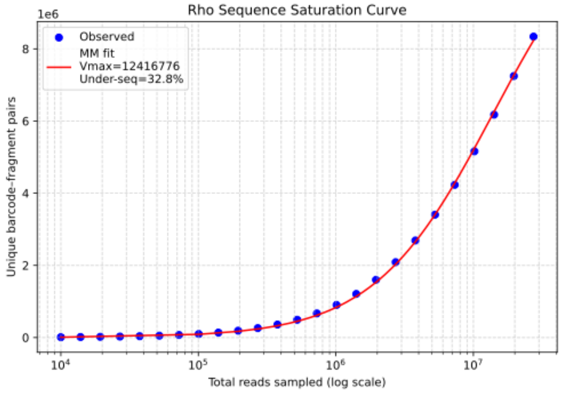

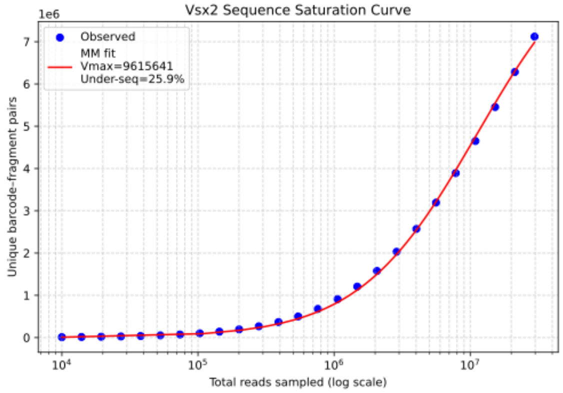

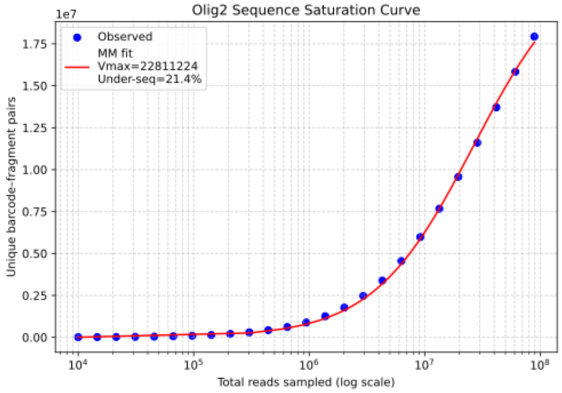

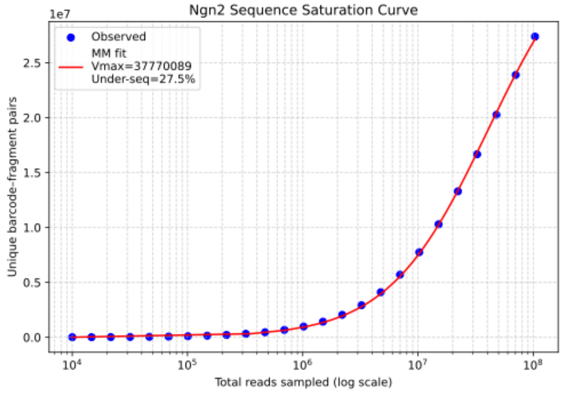

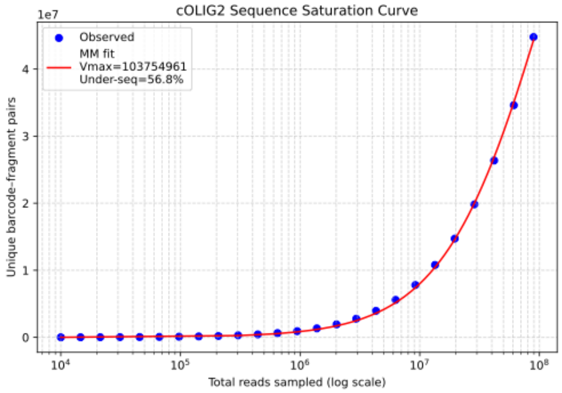

Profiled Abundance Library characteristics			
**Abundance Library**	**Fragments observed in RNA**	**Total fragments in Association Library**	**Percent profiled**
Multiplex-ExVivo-A_Cabp5	40,810	6,742,335	0.61%
Multiplex-ExVivo-B_Cabp5	32,018		0.47%
Multiplex-ExVivo-C_Cabp5	65,346		0.97%
Multiplex-InVivoP10-1_Cabp5	62,482		0.93%
Multiplex-InVivoP10-2_Cabp5	52,526		0.78%
Multiplex-InVivoP10-3_Cabp5	84,138		1.25%
Multiplex-ExVivo-A_Grm6	16,902	6,342,682	0.27%
Multiplex-ExVivo-B_Grm6	14,886		0.23%
Multiplex-ExVivo-C_Grm6	41,249		0.65%
Multiplex-InVivoP10-1_Grm6	23,755		0.37%
Multiplex-InVivoP10-2_Grm6	19,854		0.31%
Multiplex-InVivoP10-3_Grm6	43,001		0.68%
Multiplex-ExVivo-A_Rho	26,371	6,528,168	0.40%
Multiplex-ExVivo-B_Rho	18,541		0.28%
Multiplex-ExVivo-C_Rho	20,949		0.32%
Multiplex-InVivoP10-1_Rho	47,920		0.73%
Multiplex-InVivoP10-2_Rho	37,309		0.57%
Multiplex-InVivoP10-3_Rho	44,261		0.68%
Multiplex-ExVivo-A_Vsx2	9,219	5,065,113	0.18%
Multiplex-ExVivo-B_Vsx2	8,169		0.16%
Multiplex-ExVivo-C_Vsx2	23,762		0.47%
Multiplex-InVivoP10-1_Vsx2	18,753		0.37%
Multiplex-InVivoP10-2_Vsx2	14,380		0.28%
Multiplex-InVivoP10-3_Vsx2	32,321		0.64%
Olig2_LS-MPRA101	400,054	7,273,814	5.50%
Olig2_LS-MPRA202	250,985		3.45%
Olig2_LS-MPRA303	190,997		2.63%
Olig2_4hrA	223,053		3.07%
Olig2_4hrB	210,608		2.90%
Olig2_4hrC	258,610		3.56%
Olig2_12hrA	335,853		4.62%
Olig2_12hrB	323,185		4.44%
Olig2_12hrC	202,155		2.78%
Olig2_28hrA	238,341		3.28%
Olig2_28hrB	218,220		3.00%
Olig2_28hrC	212,975		2.93%
Ngn2-bc101	866,959	9,236,041	9.39%
Ngn2-bc202	754,450		8.17%
Ngn2-bc303	2,751,469		29.79%
cOLIG2_retinaA	2,441,108	11,579,155	21.08%
cOLIG2_retinaB	2,392,209		20.66%
cOLIG2_retinaC	2,557,087		22.08%
cOLIG2_retinaD	2,222,483		19.19%
cOLIG2-E2_SC1	601,082		5.19%
cOLIG2-E2_SC2	788,047		6.81%
cOLIG2-E2_SC3	1,074,630		9.28%
cOLIG2-E2_SC4	1,212,194		10.47%
cOLIG2-E4_SC1	3,719,067		32.12%
cOLIG2-E4_SC2	3,374,676		29.14%

cOLIG2-E4_SC3	2,260,800		19.52%
cOLIG2-E4_SC4	1,725,824		14.90%
