# Supplementary material for: Massively parallel reporter assay for mapping gene-specific regulatory regions at single-nucleotide resolution
Source: eLife. 2026 Feb 25;14:RP107565. doi: 10.7554/eLife.107565 (PMC12935429; doi:10.7554/eLife.107565)

Note: d-MPRA libraries were sequenced with different designated names than those in the main text. For the below source data plots:

Olig2_NR1 corresponds with the Olig2-NR2 library in the main text
Olig2_NR2 corresponds with the Olig2-NR3 library in the main text
Olig2_NR3 corresponds with the Olig2-NR1 library in the main text

Substitution Rate across CRM regions

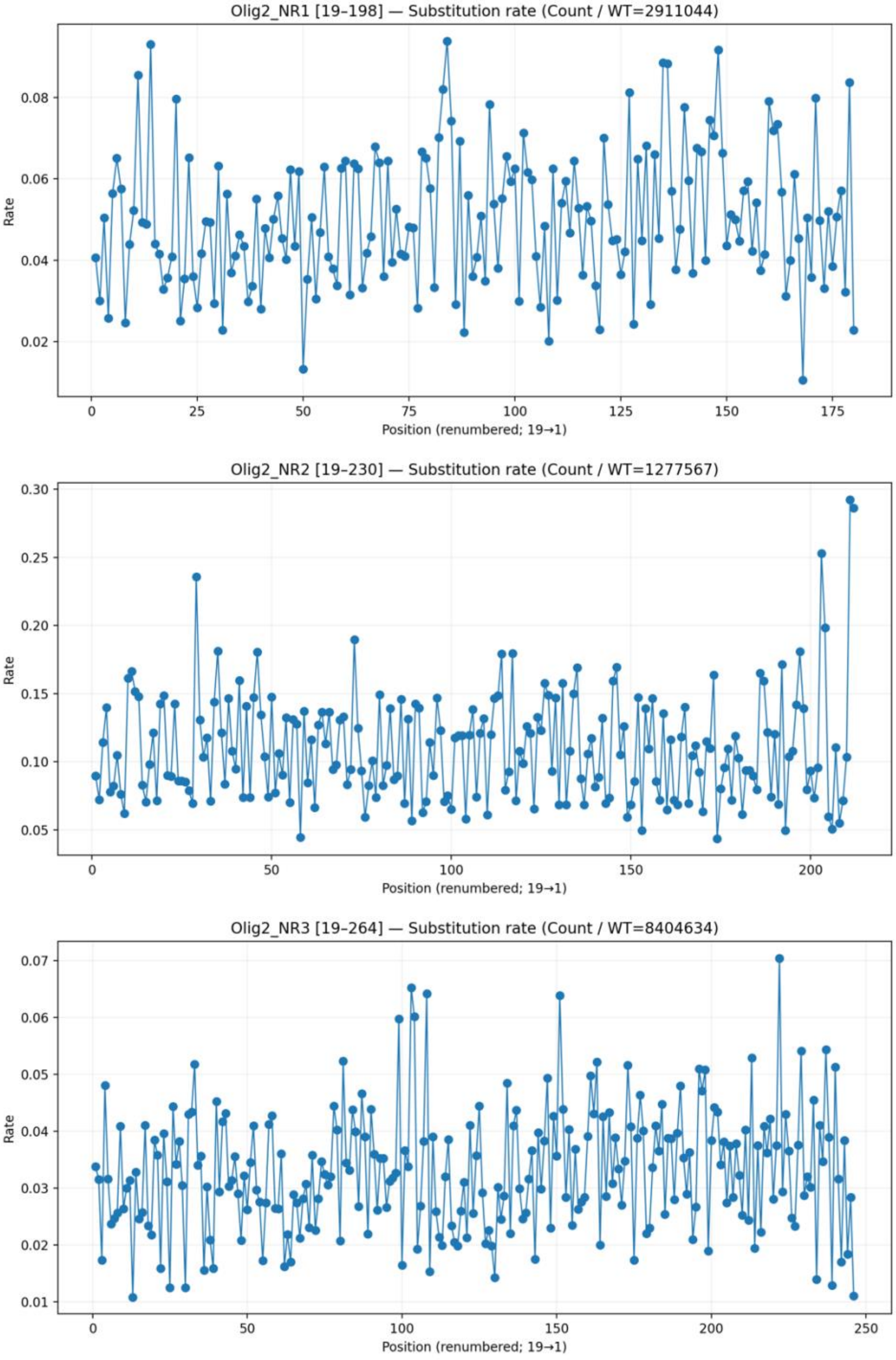

Co-occurrence of substitutions across CRM region

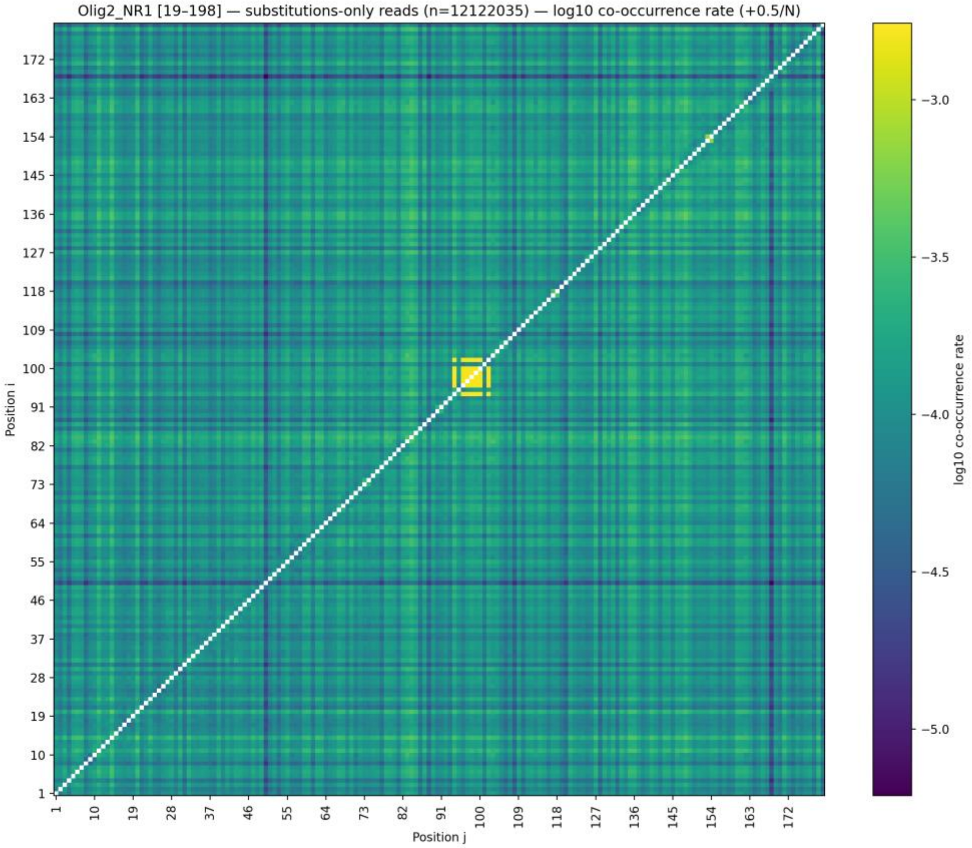

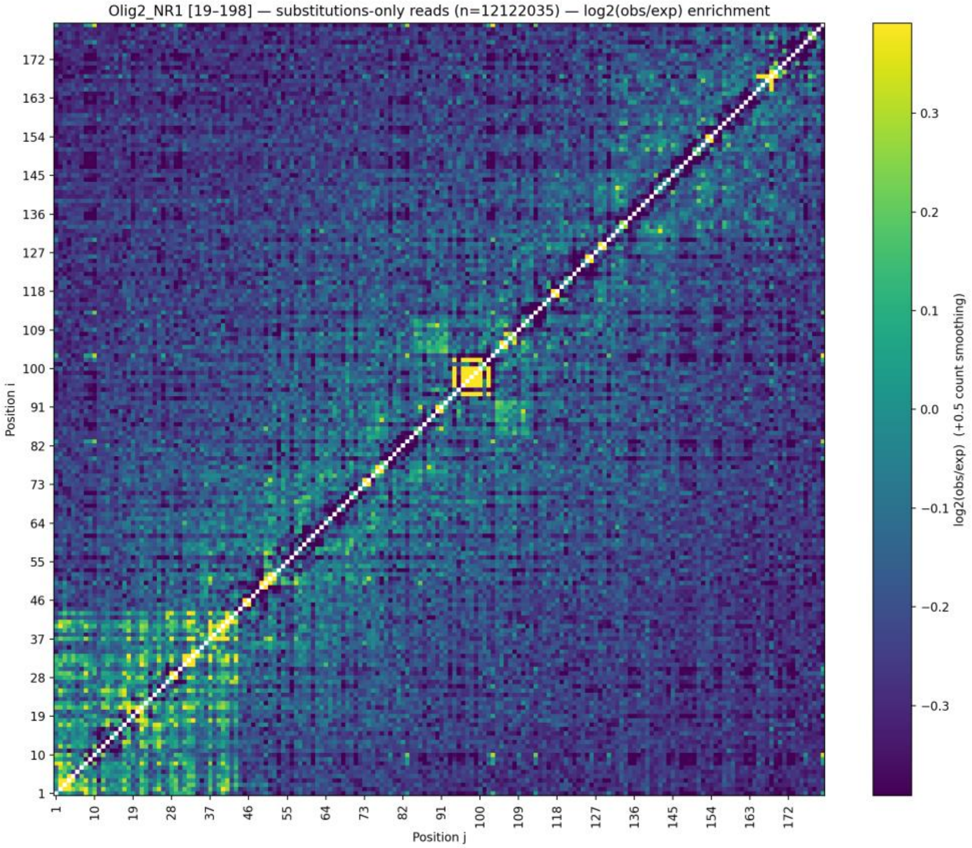

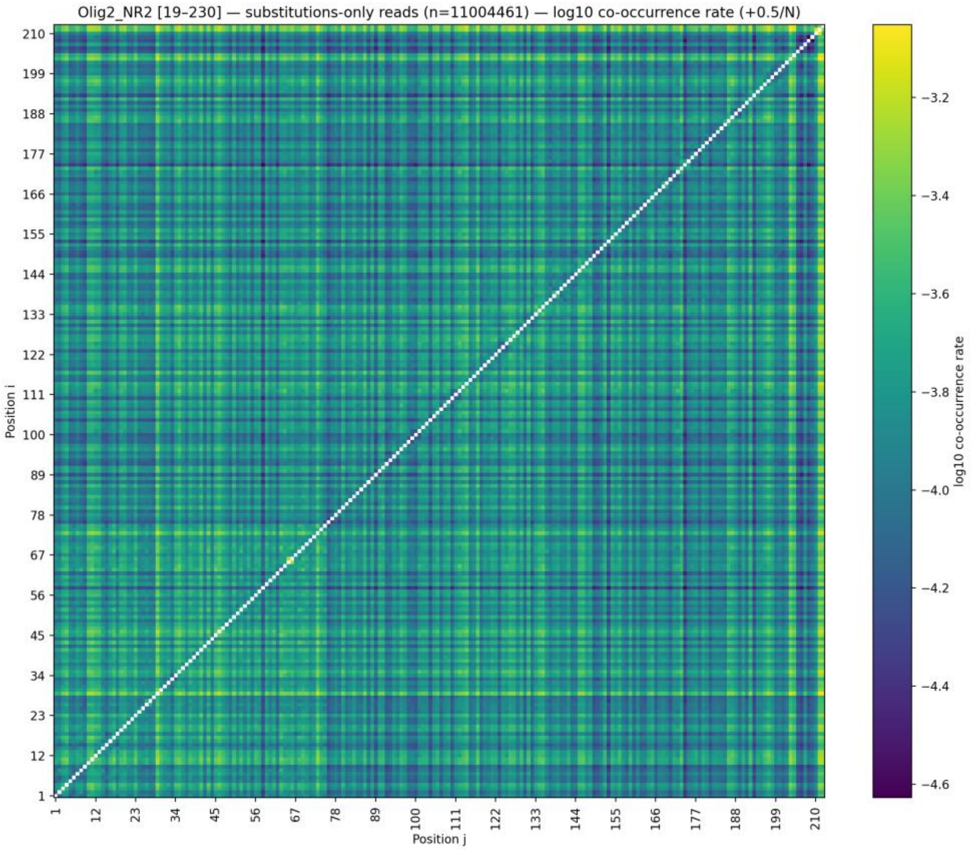

Olig2_NR2 [19–230] — substitutions-only reads (n=11004461) — log10 co-occurrence rate (+0.5/N)
Position i
Position j
log10 co-occurrence rate

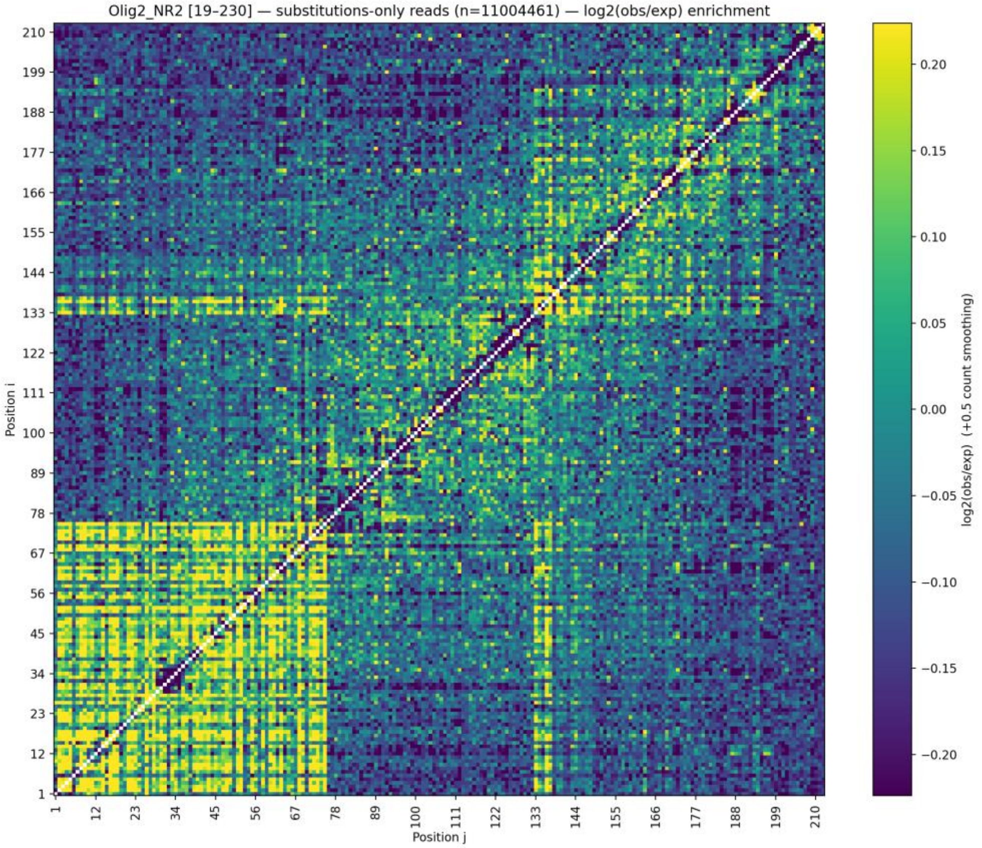

Olig2_NR2 [19–230] — substitutions-only reads (n=11004461) — log2(obs/exp) enrichment
Position i
Position j
log2(obs/exp) (+0.5 count smoothing)

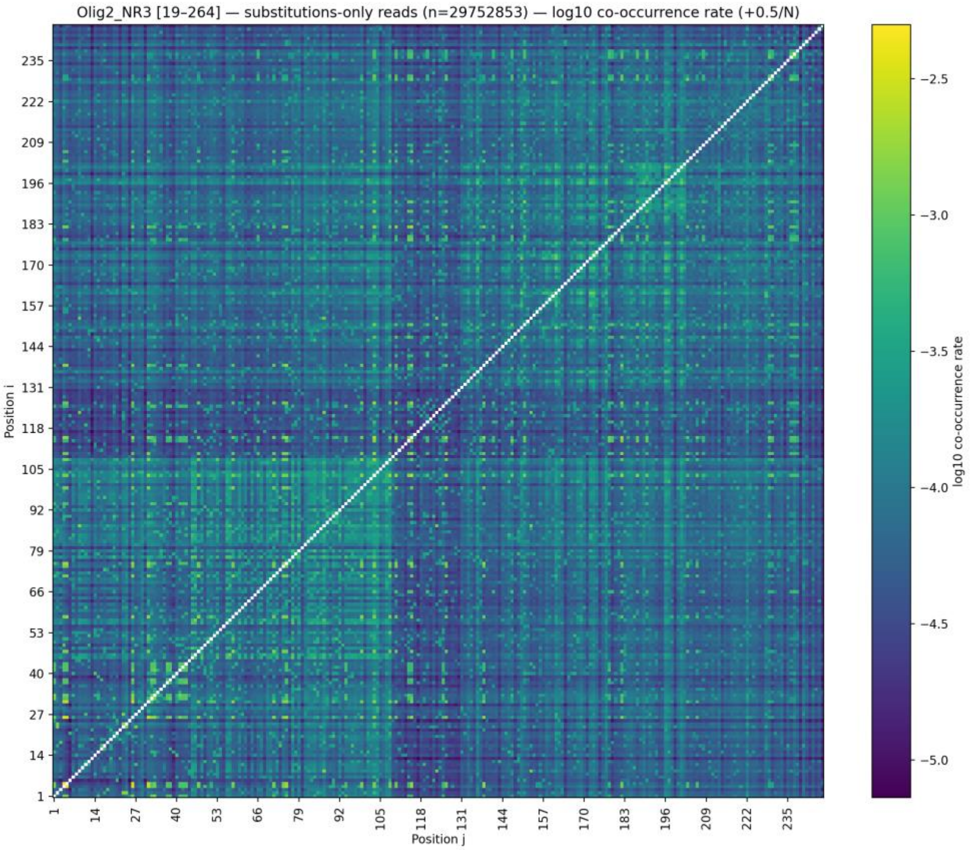
Olig2_NR3 [19–264] — substitutions-only reads (n=29752853) — log10 co-occurrence rate (+0.5/N)
Position i
Position j
1
14
27
40
53
66
79
92
105
118
131
144
157
170
183
196
209
222
235
log10 co-occurrence rate
−2.5
−3.0
−3.5
−4.0
−4.5
−5.0

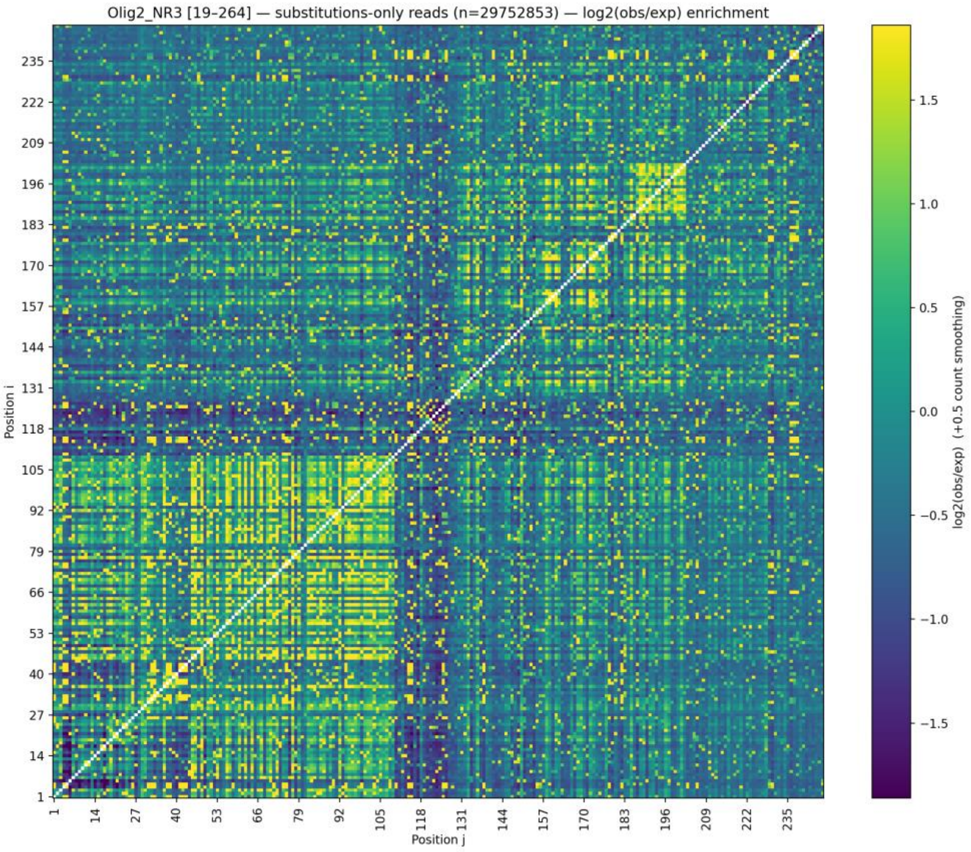
Olig2_NR3 [19–264] — substitutions-only reads (n=29752853) — log2(obs/exp) enrichment
Position i
Position j
1
14
27
40
53
66
79
92
105
118
131
144
157
170
183
196
209
222
235
log2(obs/exp) (+0.5 count smoothing)
1.5
1.0
0.5
0.0
−0.5
−1.0
−1.5

Comparison of Singleton vs Bulk analysis

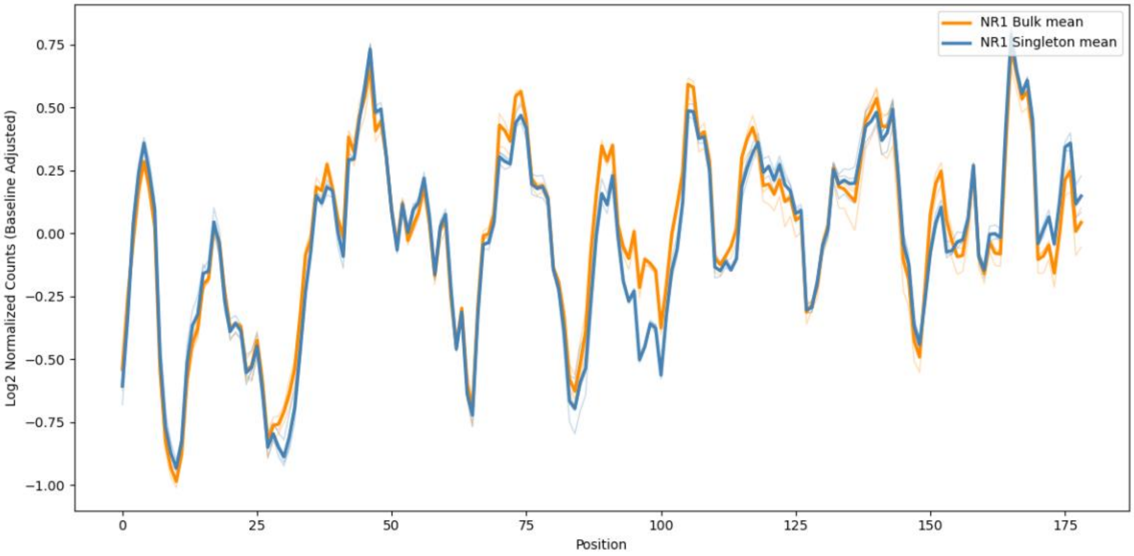

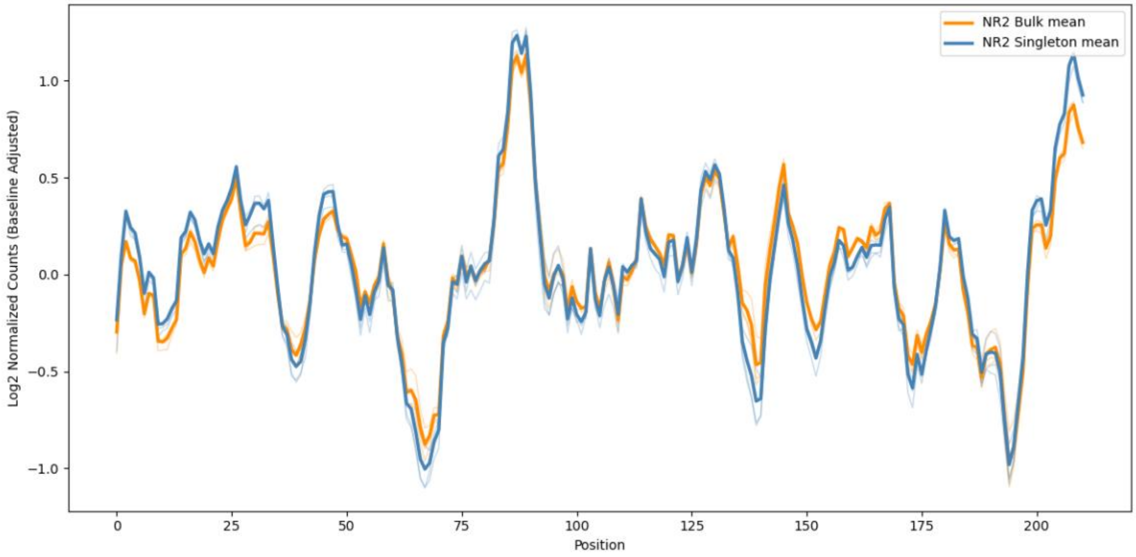

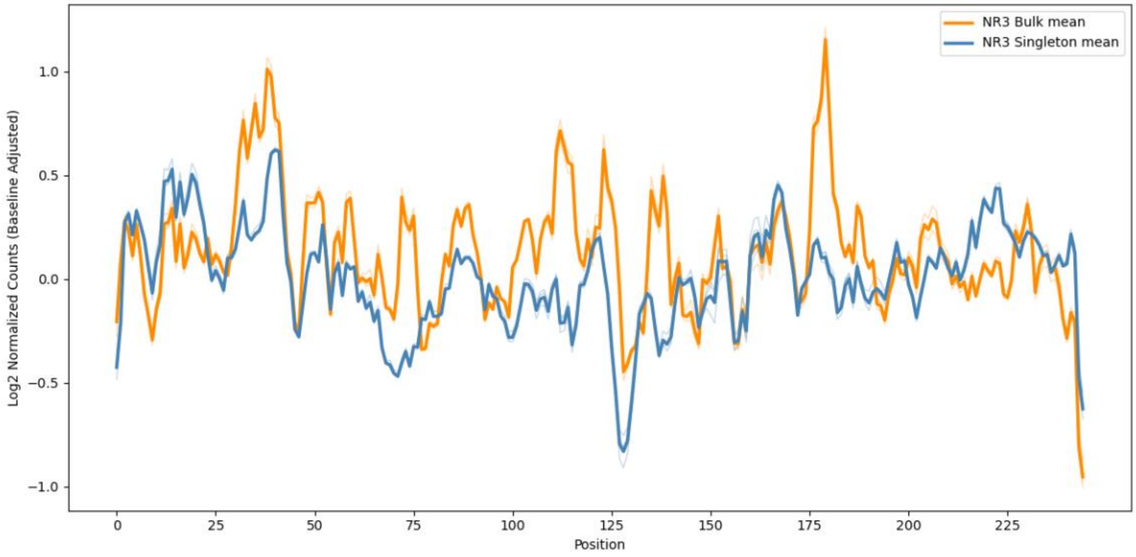

Supplement: Source data 2. [file elife-107565-data2.pdf]
